# Investigation of the motor skills assessments of typically developing preschool children in China

**DOI:** 10.1186/s12887-021-03098-w

**Published:** 2022-02-11

**Authors:** H. Q. Song, P. W. C. Lau, J. J. Wang

**Affiliations:** 1 Department of Sport, Physical Education & Health, Hong Kong Baptist University, Hong Kong, China; 2 National Fitness Research Center, China Institute of Sport Science, Beijing, China

**Keywords:** motor skills assessment tools, pre-school children, Chinese self-constructed tools

## Abstract

**Background:**

The assessment of preschoolers’ motor skills is essential to know young children’s motor development and evaluate the intervention effects of promotion in children’s sports activities. The purpose of this study was to review the motor skills assessment tools in Chinese pre-school-aged children, compare them in the international context, and provide guidelines to find appropriate motor skill assessment tools for developing children in China.

**Methods:**

A comprehensive literature search was carried out using the WANFAGN, CNKI, VIP, ERIC, EMBASE, MEDLINE, and SPORT Discus databases. Relevant articles published between January 2000 and May 2020 were retrieved. Studies that described the discriminative and evaluative measures of motor skills among the population aged 3–6 years in China were included.

**Results:**

A total of 17 studies were included in this study describing seven tools, including four self-developed tools and three international tools used in China. TGMD-2 appeared in a large proportion of the studies. The international tools used in China were incomplete in terms of translation, verification of reliability and validity, item selection, and implementation. Regarding the self-constructed tools, the CDCC was the most utilized self-developed tool, but it was mainly applied in intellectual development assessment. By comparing Chinese self-constructed and international tools, the construction of the CDCC and the Gross Motor Development Assessment Scale contained relatively complete development steps. However, the test content, validity and reliability, implementation instruction, and generalizability of self-constructed tools are still lacking.

**Conclusions:**

Both international and self-developed motor skills assessment tools have been rarely applied in China. Available tools lack enough validation and appropriate adjustments. Cultural differences in motor development between Chinese and Western populations should be considered when constructing a Chinese localized motor skill assessment tool.

## Background

Motor skills are the cornerstone for humans to partake in various physical activities (PA) [1], which can reflect the development level of the neuro-motor system, physical fitness, body mass index, cognitive competence, and other growth outcomes in children [1–3]. Pre-school years are identified as a crucial time in terms of forming and developing various motor skills. During this period, children learn a group of fundamental motor skills and apply them to organized and non-organized PA, which plays a critical role in sports participation when they grow up [4]. A study has shown that children with poor motor skills are more likely to be unconfident and at risk of being overweight and obese than children with good motor skills [5]. Furthermore, if motor skill problems in early childhood are not identified and remedied in time, it may affect their motor skill in adulthood and lead to lifetime motor skill problems [6, 7].

It is necessary to accurately assess the level of motor skills in early childhood, for which valid and reliable assessment tools must be designed for preschoolers. Motor skills assessment tools (MSAT) such as Bruininks-Oseretsky Test of Motor Proficiency, second edition (BOT-2) can identify children with motor problems [8]. In addition, the MSAT can evaluate interventions aimed to promote PA in pre-school children. For example, after complimenting a 10-week PA program, all components of the Test of Gross Motor Development second edition (TGMD-2) scores were significantly improved (P < 0.001) among the participants [9].

Currently, 13 MSAT for pre-school children are widely used because of their detailed assessment description, clear scoring method, and short assessment time (see Table 1) [8, 10–21]. These tools were developed between 1974 and 2018, and all are designed in western. Some MSAT is aimed at healthy children, and others are designed for children with motor impairment. The majority of the assessments have specific subscales; only three instruments have no sub-items. In terms of the number of items, Körperkoordination-Test für Kinder (KTK) [12] has the least number of items (four items) whereas Peabody Developmental Motor Scales, second edition (PDMS-2) [18] has the most number of items (249 items). The assessment time varies from 10 to 60 minutes. Raw scores of MSAT are usually converted into subscale scores, total scores, and percentile scores to demonstrate young children’s motor skills competency.

**Table 1:** Characteristics of motor skills assessment tools

Test tool	Author and date	Country	Age (year)	Type	Subscales	Number of items	Assessment time	Raw score conversion
Athletic Skills Track (AST-1)	Wormhoudt et al. (2012) [10]	Netherlands	4-6	Fundamental movement skills	NA	5	NA	Time to complete the track
Bruininks–Oseretsky Test of Motor Proficiency (BOTMP)	Bruininks (1978) [8]	USA	4 years 6 months-14 years 6 months	Motor proficiency	Fine motor precision, fine motor integration, manual dexterity, bilateral coordination, balance, running speed and agility, upper limb coordination, strength	46	40-60	Subscale scores, total score, standard score, percentile score
CHAMPS Motor Skills Protocol (CMSP)	Williams, et al. (2009) [11]	USA	3-5	Motor skills status	Locomotor skills, object control skills	12	45	Subscale scores, total score, distraction scores
Körperkoordination-Test für Kinder (KTK)	Kiphard & Schilling (1974, 2007) [12]	German	5-14	Gross motor coordination	NA	4	20	Standardized motor quotients, percentile score
Movement Assessment Battery for Children (MABC)	Henderson & Sugden (1992) [13]	USA	4-12	Motor impairment	Manual dexterity, ball skills, static & dynamic balance	8	20-30	Impairment scores, cut-off scores, percentile score
Movement Assessment Battery for Children-2nd edition (MABC-2)	Henderson, Sugden, & Barnett (2007) [14]	USA	3-16	Motor impairment	Manual dexterity, aiming and catching, balance	8	20-40	Standard scores, total score, percentile score
Motor-Proficiency-Test for children between 4 and 6 years of age (MOT 4-6)	Zimmer & Volkamer (1987) [15]	German	4-6	Gross and fine motor skills	Locomotor skills, object control skills, stability, fine motor skills	18 (1 practice item)	15-20	Standardized motor quotients, percentile score,
Motor Skill Checklist (MSC)	Peersman, et al. (2011) [16]	Netherlands	3-5	Motor skills (questionnaire by teachers)	NA	28	NA	Percentile score, total score
Preschool Child Development Inventory (PCDI)	Gudmundsson & Gretarsson (1993,1997,2009) [17]	Iceland	3-6	Motor and language skills (questionnaire by mothers)	Gross motor skills, fine motor skills, self help	10	NA	T-score, standard score
Peabody Developmental Motor Scales, second edition (PDMS-2)	Folio & Fewell (2000) [18]	USA	0-5 years and 11 months	Early motor milestones and fundamental movement skills	Reflexes (only for children below one year), static balance, locomotion, ball skills, grasping, visual-motor integration	249	45-60	Age equivalent, percentile score, standard score, gross motor quotients, fine motor quotients, total motor quotients, z-score
Test of Gross Motor Development, second edition (TGMD-2)	Ulrich (2000) [19]	USA	3-10	Fundamental movement skills	Locomotor skills, object control skills	12	15-20	Standard score, percentile score, subscale scores, total score
Test of Gross Motor Development, third edition (TGMD-3)	Ulrich (2013) [20]	USA	3-10	Fundamental movement skills	Locomotor skills, ball skills	13	10-15	Subscale scores, total score
Zurich Neuromotor Assessment second edition (ZNA-2)	Kakebeeke, et al. (2018) [21]	Switzerland	3-18	Motor proficiency	Fine motor tasks, pure motor tasks, static balance, dynamic balance	11	20-30	Subscale scores, total score, z-score, standard score

Researchers have already compiled the literature about MSAT in meta-analyses and systematic reviews [22–25]. In China, the importance of motor skill proficiency for young children has been realized, but more attention is paid to the motor skills in children with disabilities [26–30]. Recently, a few studies summarizing MSAT for young children emerged in China [31–33]. However, these reviews presented general features of MSAT used in the international context, ignoring how these tools were used among Chinese children. Although international assessment tools are widely used for generalization and acceptability, self-constructed tools for Chinese preschoolers in China deserve attention due to the different cultural and parenting practices. Given that there is little information about MSAT in China, the adoption of international assessment tools and self-construction tools needs further investigation to ensure its validity, reliability, and effectiveness. Therefore, the assessment results can be comparable to the international studies.

The first aim of this study is to review the features of prevalent MSAT used in China, including international and self-constructed assessment tools, among typically developing pre-school children aged 3–-6 years. Secondly, the scientific standard procedures for the development of internationally recognized tools are integrated into this study as a reference to evaluate developing steps of the self-constructed assessment tools. Finally, recommendations will be made for the adopted international and self-constructed tools to modify their application among 3–6 years old Chinese pre-school children.

## Method

This review was conducted following the guidelines from Preferred Reporting Items for Systematic Reviews and Meta-Analysis (PRISMA) [34].

### Search strategy and selection process

The databases WANFAGN, CNKI, VIP, ERIC, EMBASE, MEDLINE, and SPORT Discus, were searched manually. The publication dates were between 1 January 2000 and 1 May 2020. The search terms were divided into three categories:

1) “3–6-year-old child,*” including“preschoolers,*” “pre-school child,*” “younger child,*” “kindergarten child,*” “kindergarten,*” “child care center,*” and “early childhood.”

2) “Motor skill, *” including “movement skill,*” “motor development,” “gross motor skill,*” “fine motor skill,*” “motor performance,” “motor function,*” “motor ability,*” “motor competence,” and “fundamental motor skill.*”

3) “Assessment tool, *” including “test tool,*” “test batter,*” “assessment scale,*” “evaluation system,*” “measurement,*” “field-based testing protocol,*” “scale,*” “test,*” and “measurement tool.*”

An asterisk followed some terms, which indicated that plural forms of certain words were searched as well. Whenever possible, related terms were searched for within the databases.

The literature search included four stages. Firstly, all search terms were searched simultaneously using the Boolean calculation words “AND” “OR” to connect the search terms for computer search. Secondly, the authors imported all the retrieved results to the software EndNote X9. Next, they screened for titles and abstracts that potentially met the inclusion criteria and would be appropriate for the full-text copy. Then, all duplicates and unrelated studies were removed from the software. Then the full-text articles were checked for their eligibility by an independent researcher. Finally, the authors used a snowball strategy in addition to the articles included in the initial search. They examined the reference lists of the retrieved articles for potential new articles that were eligible and pearled to ensure no valuable studies were missed. The authors (Lau PWC, Wang JJ, and Song HQ) discussed any discrepancies until a consensus was reached.

### Inclusion and exclusion criteria

Studies meeting the following criteria were included in this review.

1. Language limitation

Only articles published in English or Chinese (traditional and simplified visions) were considered.

2) Access to articles

Studies were included as full-text peer-reviewed journal articles available online. Dissertations, conference papers, and textbooks are excluded from this search.

3) Setting

Studies focusing on Chinese children's motor ability data were included.

4) Population

Any study about the age range or mean age of typically developing children from three to six years was retrieved. Studies for children with intellectual or developmental delay, disabilities, chronic diseases, and any other health problems, such as cerebral palsy, autism, and ADHD, were excluded. However, after sorting out the articles, we found that some studies sampled children from pre-school and primary school together. We analyzed whether children were divided into age groups to separate the preschoolers from other younger or older children in these cases. If so, the study would be included, and the related information about preschoolers was presented. Conversely, when the authors used the whole sample without age bands for analysis, if there were results concerning the single age included in 3–6 years, e.g., 3–10 years with individual results about four years, it can be considered a pre-school sample. But if the sample age was beyond the age range without isolated age results, e.g., 5–10 years, the studies were not considered.

5) The motor skills assessment tool

Because there are different definitions and classifications for motor skills, variations of MSAT, including motor skill questionnaire, product-oriented assessment tools, or process-oriented assessment tools of at least one gross motor skill, or fine motor skills or other subscales (e.g., stability, balance), detailed test tools, and all evaluative measures mentioned in the study were included.

6) Article type

Cross-sectional studies, longitudinal studies, and intervention studies describing MSAT were included. Reviews about pre-school children’s motor skill test tools were excluded.

### Data extraction

The author and date, district, participant description, test tool, settings, testers, use of items, and reliability and validity were extracted and summarized in Tables 2 and 3.

**Table 2:** International assessment tools in China

Test tool	Author and date	District	Age	Sample size	Male proportion (%)	Setting	Setting number	Testers	Version	Use of items	Reliability	Validity
MABC-2	Hua et al. (2013) [35]	Suzhou	3-6	1823	50.2	1	10	42T	Chinese version	All items	Inter-rater: 0.892 to 0.998; Test-retest: 0.830 to 0.985; Internal: 0.502	Content: average I-CVI was 0.985; Criterion-related: 0.750 (MABC-2 manual dexterity and PDMS-2 fine motor subsets); Factorial: 0.34 to 0.77
TGMD-2	Li (2009) [36]	Jinan	3-10	511	50.9	2	NA	T	NA	All items	NA	NA
	Mo (2015) [37]	Hangzhou	3-6	108	49.1	1	3	NA	NA	All items	NA	NA
	Jia (2015) [38]	Guangzhou	4-6	47	51.1	1	1	NA	NA	All items	NA	NA
	Ning et al. (2016) [39]	Xi’an	4-6	614	54.1	1	6	NA	NA	All items	Composite: 0.75 in locomotor and 0.67 in object control subscale	Convergent: 0.56; Discriminant: 0.53 in locomotor and 0.62 in object control subscale
	Dai et al. (2017) [40]	Shanghai	3-6	206	50.5	1	3	NA	NA	R, H, K, SD, HJ, C	NA	NA
	Chen (2017) [41]	Zhangzhou	3-6	120	NA	1	1	T	NA	Object control subscale	NA	NA
	Chen & Yu (2018) [42]	NA	3-6	144	NA	1	1	NA	NA	HJ, R, H, C, SD, UR	NA	NA
	Liu (2018) [43]	Shandong	3-6	788	60.3	1	3	T	NA	All items	NA	NA
	Jin (2019) [44]	Shanghai	5-6	50	NA	1	1	NA	NA	NA	NA	NA
TGMD-3	Diao et al. (2018) [45]	Shanghai	3-10	1118	50.0	2	24	T	NA	All items	Inter-rater: 0.983; Test-retest: 0.974; Internal consistency: 0.81	Construct validity: NNFI=0.947, CFI=0.957, GFI=0.956, RMSEA=0.049, SRMR=0.039; Cross-group and stability validity: CFI and NNFI> 0.93, RMSEA<0.05

Setting: 1=kindergarten/day-care centers, 2= primary schools and kindergartens; *Testers T* trainees, *CT* class teachers, *Use of items*: *C* catching, *H* hopping, *HJ* horizontal jumping, *K* kicking, *R* running, *SD* stationary dribbling, *UR* underhand rolling, *NA* not available

**Table 3:** New developed assessment tools in China

Test tool	Author and date	District	Age	Sample size	Male proportion (%)	Setting	Setting number	Subscales	Number of items	Assessment time	Raw score conversion	Instruction	Reliability	Validity
Children’s Developmental Centre of China Scale (CDCC)	Zhou & Zhang (1994) [46]	Mainland	3-6	2368	NA	2	NA	Intellectual development, motor development	16	NA	TS, PS, SS	Manual instruction	Internal consistency: 0.708 to 0.953; Test-retest: 0.893	Content: high; Construct: relatively high; Criterion-related: 0.603 (Standdord-Bibent Test of Intelligence and CDCC)
	Chen & Li (2015) [47]	Zhejiang	5-6	966	50.3	3	90	Intellectual development, motor development	16	NA	TS	NA	NA	NA
	Chen et al. (2013) [48]	Zhejiang		990	50.3	3	91	Motor development	5	NA	TS	NA	Internal consistency: 0.781	NA
Gross Motor Development Assessment Scale	Guo et al. (2018) [49]	Beijing	3-6	280	50.0	3	4	Locomotor, object control, posture control	10	15 min	TS, SS	Reference version	Inter-rater: 0.954 to 0.988; Test-retest: 0.926 to 0.997; Homogeneity: 0.837 to 0.861	Construct: 0.607 to 0.890; Convergent and discriminant validity: 0.316 and 0.923
Athletic Ability Test Scale of Urban Community Children	Zhou (2018) [50]	Beijing	3-6	60	NA	1	NA	Balance coordination and agility, strength, manual dexterity	12	NA	AS, TS	NA	NA	NA
Evaluation System of Sports Ability	Guo & Zhou (2018) [51]	Wuhan	3-6	200	NA	3	3	NA	11	NA	NA	NA	Scale: 0.732 to 0.824	NA

Setting: 1=community, 2=national program, 3= primary schools and kindergartens, 4= health center, *Raw score conversion*: *AS* average score, *PS* percentile score, *SS* subscale scores, *TS* total score, *NA* not available

## Results

**Fig. 1:**
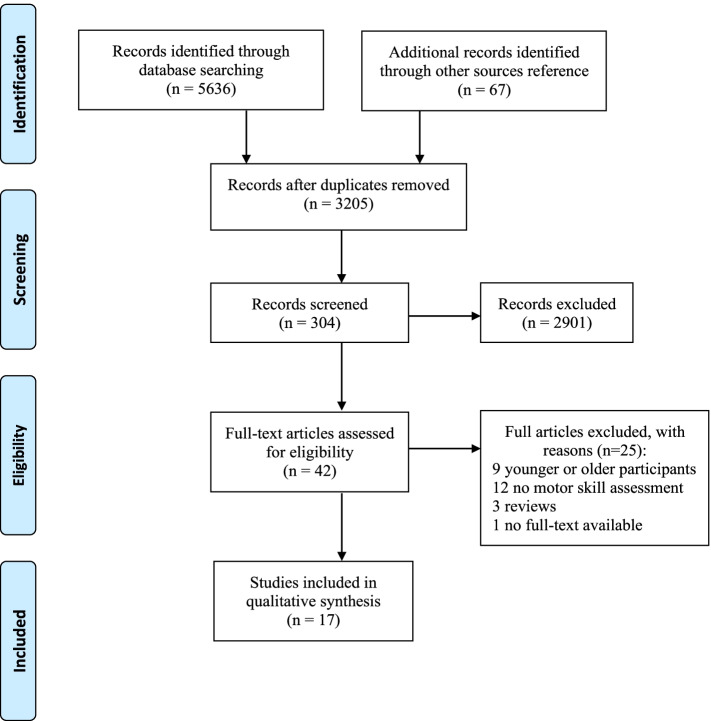
PRISMA flow diagram of study selection process

The initial search produced 5,636 articles, which was reduced to 304 after abstract and title screening and removal of duplicates. A total of 42 full articles were reviewed, and a further 25 articles not meeting the inclusion criteria were excluded. Thus, in total, 17 papers were eligible for this review (see Figure 1).

### Features of prevalent MSAT in Chinese preschoolers

#### International assessment tools adopted in China

Eleven of 17 studies described the current situation of motor skill assessments in China by using international tools [35–45]. Three uniquely recognized MSAT were identified across the 11 studies. The Test of Gross Motor Development, second edition, (TGMD-2) was used in nine studies. The Test of Gross Motor Development, third edition, (TGMD-3), and the Movement Assessment Battery for Children, second edition, (MABC-2) were used in one study respectively. Of the included studies, data from 5,529 children from 53 childcare centers were incorporated. Thus, studies included between 47 and 1,823 participants from 1 to 24 childcare centers. Motor skills tests were most commonly conducted at the pre-school site (nine studies); only two were carried out in primary schools and kindergartens. Below are the major features demonstrated (See Table 2).

(A) Test translation

In terms of the translation of the tools, only Hua showed information about the translation of the MABC-2, and an independent translator back-translated the information [35]. About half of the studies described the recruitment and training process of testers. Li only mentioned that specially trained testers rated the participants’ motor performance according to scoring criteria [36]. The other studies showed training details, including testing process, instrument items, scoring standards, safety management, and practice assessment [35, 41, 43, 45].

(B) Test item selection

Of nine articles that used the TGMD-2 in China, two-thirds used all original items [36–39, 43], one-third of them used partial items from the instrument [40–42]. Dai selected running, hopping, kicking, and stationary dribbling as the easiest and most difficult items from the TGMD-2. Horizontal jumping and catching were used to estimate the coordination skills of limbs and joints [40]. To explore the effect of ball game skills on object control skills in children, Chen used all items in the object control skills subscale [41]. In another study, Yu and Chen included horizontal jumping, running, hopping in locomotor skills subscale and catching, stationary dribbling, and underhand rolling in object control skills subscale, without explaining why and how the six items were determined [42]. Moreover, no study adjusted items according to the condition of local children.

(III) Reliability and validity issue

Three studies verified both the reliability and validity of international tools [35, 39, 45]. Composite reliability score of locomotor skills and object control skills subscale of the TGMD-2 was 0.75 and 0.67 respectively. To determine the amount of convergence, Pearson's r was calculated and demonstrated the correlation between subscales was 0.56. Besides, the average variance extracted values were higher than the Pearson correlation coefficient, which suggested that subscales of the TGMD-2 had discriminant validity [39]. ICCs of “walking heels raised” and “drawing trail” in the MABC-2 were good but below 0.9, and if the items “walking heels raised” and “drawing trail” were removed, Cronbach’s alpha coefficient would increase. Moreover, the correlation between components of the MABC-2 and the PDMS-2 was high to weak. These findings indicated that the reliability and validity of age band 1 (3–6 years old) of the MABC-2 were fair [35]. Internal consistency reliability (0.81, Cronbach’s Alpha), test-retest reliability (r = 0.974), and inter-rater reliability (r = 0.983) were good; the construct validity was acceptable, indicating that the TGMD-3 was an appropriate tool [45].

(IV) Norm establishment

Among the three international tools, only the TGMD-3 has norms in Shanghai, and the establishment process is complete [45]. The stratified sampling method was adopted to select 12 kindergartens and 12 primary schools with 1,118 children from both urban and rural environments. The difficulty (0.63) and discrimination (0.4) of the TGMD-3 were appropriate. Validity and reliability were examined. Significant gender and age differences were found in the ball skills subscale and significant age differences in the locomotor skills subscale and total scale of the TGMD-3. Thus, the norm of the ball skills subscale was established by gender and age, and norms of the total scale and locomotor skills subscale were established by age. The original score was converted into a Z score linearly. According to the rule of the Z score, the raw scores of the two subscales and the total scale were converted into the 1–5 level norm.

(E) Gender, age, and urban/rural differences

Eight studies showed that the competence of fundamental movement skills of pre-school children increased with age, which means that older children grasped motor skills better [36–43]. Mo and Dai found no gender difference in total and subscale scores [37–40]. In contrast, Ning and Liu observed that boys performed better than girls in gross motor skills [39, 43]; Jia and Liu found that boys were better at controlling balls and girls at locomotor skills [38, 43]. In China, only Liu observed that urban children got higher scores in object control skills than rural children, especially in kicking and overhand throwing [43].

Only three articles examined the reliability and validity of tools, and one mentioned the translation of the tool. The TGMD-2 was the most frequently used international MSAT in China. Two-thirds of studies applying TGMD-2 chose to use all items of the original tool, and one-third selected some of the items for specific test purposes.

#### Chinese self-constructed assessment tools

A total of six studies among the included articles described four MSAT developed by Chinese researchers [46–51]. These articles were published between 1994 and 2018. Children were recruited from the community, health care centers, and pre-schools. The sample size of included studies varied from 60 to 2,368 children (See Table 3).

(A) Children’s Developmental Centre of China Scale (CDCC)

The CDCC measures the development of Chinese children aged 3–6 years based on their psychological features. This test can be used to provide indicators of early childhood development for workers in pre-school education, health care, and pediatrics to facilitate education, assessment, and intervention. The CDCC was initially developed with 22 items in four subtests by combining the psychological development tests with the practical experience of early childhood educators. First, a preliminary test was carried out on 500 children in six Chinese cities. After statistical processing and summary, 16 items were extracted to form the scale. Next, a pilot test was conducted on 2,368 children in 18 cities in China, and the sample was stratified by age, gender, parental education level, and residence [46]. By analyzing the pilot test results, the test sequence and content of test items were adjusted, and the scale standardization was completed. Then the CDCC was field-tested nationwide to assess its feasibility and further modified according to the problems that originated from the pilot test results.

The CDCC consists of 11 items in the intellectual development subscale and five items in the motor development subscale. The motor development subscale includes four items to assess gross motor skills and skills to quickly pick up beans to measure fine motor skills. Each item in the motor development subscale is rated on a six-point rating scale from 0 (weakest performance) to 5 (best performance). Total score, percentile score, and subscale scores are used to express the development level. The CDCC has good reliability and validity. The internal consistency reliability ranges from 0.708 to 0.953, ICC of test-retest reliability was 0.893, and validity was high. Chen and Li found that children’s CDCC score (41.88 ± 28.76) was significantly related to corner activity [47]. Chen et al. used the motor development subscale of CDCC and investigated that the relationship between children’s motor development and outdoor activity quality was weak [48].

(B) Gross Motor Development Assessment Scale

The Gross Motor Development Assessment Scale is a quick and convenient test to measure gross motor skills, which creates the opportunity for evaluation and guidance of the current situation of pre-school children’s motor development. Originally, 17 motor skills assessments were extracted to generate 34 items from three subtests. Then a questionnaire with 34 items compiled according to the Likert Scale five-point scoring method was sent to experts for their opinions. Ten items were selected, and scoring criteria were determined to form the first draft. A pilot test was conducted on 56 children to test the acceptability and universality of the first draft. According to the statistical properties results, ten items (below) were included in the final draft.

The scale is divided into locomotor skills, object control skills, and posture control skills subscales. The locomotor subtest consists of four items: running, hopping, horizontal jumping, and leaping. The object control part consists of four items: kicking, catching, one-hand throwing, and stationary dribbling. The posture control subtest consists of two items: one-leg balance and balance beam. A five-point scale from 0 to 4 is used to score the motor performance on each item. It takes about 15 minutes to administer the test. The inter-rater reliability (ranging from 0.954 to 0.988), test-retest reliability (ranging from 0.926 to 0.997), and homogeneity reliability (ranging from 0.837 to 0.861) of the Gross Motor Development Assessment Scale were good. In addition, the construct validity (ranging from 0.607 to 0.890), convergent validity, and discriminant validity were acceptable. Guo found no significant gender difference in subscale scores, while there were significant differences in subscales in different age groups. Therefore, in the establishment of norms, different criteria are required for each age group. However, only the reference version of evaluation standards established on 280 children is available [49].

(III) Athletic Ability Test Scale of Urban Community Children

The Athletic Ability Test Scale of Urban Community Children is designed to assess motor skills in 3–6 years old children in the urban community [50]. The scale is established through the discussion by experts in the field of pre-school education and physical education. It contains three subscales: balance coordination and agility (five items), strength (five items), and manual dexterity (two items). The highest score for each item is 5, and the lowest score is 1. Raw scores are converted into the total score and average score. Boys and girls in the same age group use the same scoring criteria. The test requires equipment such as sandbags and balls. Before the formal test, 12 children were primarily tested to verify the reliability and validity of the scale. Regrettably, no specific information about reliability, validity, and item selection was reported. Sixty children in Beijing participated in the test. Results suggested that the development of balance and coordination, agility, manual dexterity was good, the development of strength and endurance, especially for upper body strength, was not satisfactory. There was no significant gender difference in the total score, but boys performed better in balance, bounce, and endurance than girls. Girls performed better in manual dexterity.

(IV) Evaluation System of Sports Ability

The Evaluation System of Sports Ability is suitable for assessing fundamental movement skills for children aged three to six years. The test items and measurement were generated through two rounds of questionnaires, and items deemed important were retained. The test includes 11 items: 20 meters, standing long jump, walking on hands and feet, five meters back and forth, balance beam, sandbag throw, a ball bouncing, fast run, standing on one foot, skipping rope, and arms support. The scoring criteria are established by the percentile method and T standard score conversion method. Scale reliability of the test ranges from 0.732 to 0.824. Guo and Zhou found no significant gender difference, whereas there were significant differences in terms of age [51].

Among these four self-constructed MSAT, two tools are used to assess gross motor skills, and two measures fine and gross motor skills. Although the CDCC was the most utilized test tool in China, its intellectual development assessment was applied more than its motor development assessment. Therefore, current studies involving the motor development subscale were aimed to explore the relationship between motor development and physical activity. Except for the Athletic Ability Test Scale of Urban Community Children, all other self-constructed tools in China verify their reliability and validity.

### Evaluation on developing steps of the self-constructed tools in China

The construction of one tool needs a scientific and standard procedure. Therefore, this review summarized and integrated the development steps of international tools such as MABC-2, the TGMD-2, and the Communication Assessment Tool (CAT) and compared the development steps of Chinese self-constructed tools with those of international tools [14, 19, 52]. According to the international tools, there are six steps, including “original item selection,” “establishment of scoring criteria,” “pretest of initial items,” “determination of final items,” “reliability and validity examination,” and “field test to assess feasibility” in developing an instrument. Each step contains a detailed description (See Table 4).

**Table 4:** Evaluation on developing steps of the self-constructed tools in China

Steps to conduct an instrument	Description	Athletic Ability Test Scale of Urban Community Children	Children’s Developmental Centre of China Scale (CDCC)	Evaluation System of Sports Ability	Gross Motor Development Assessment Scale
Original item generation	Literature review of well-developed instruments according to the test purpose.		√	√	√
	Consult with experts from related field to identify the factors for instrument in target population.	√	√	√	√
	Compile the items suitable for factors through review into a questionnaire and send it to experts for comments.	√	√	√	√
Establishment of scoring criteria	Establishing scoring criteria for each item, in which the assignment details can be found.	√	√	√	√
Pretest of initial items	A small group of target population are invited as the pilot test sample for preliminary examination of statistical properties (e.g., difficulty and suitability) in items. The group should include a relatively equal proportion of males and females, a diversity of ages and racial/ethnic backgrounds.		√		√
	Videotape test to facilitate analysis of participants’ response to items and ideas for new items.				√
	Analyze the variance coefficient, completion rate and relationships between each item and its subscale to delete or retain items.		√		√
Determination of final items	Selection of response scale				
	Experts are invited to write response scale to gauge the importance of each item. The items deemed “very important” will be retained.				
	Addition of items to ensure the comprehensiveness after careful review of the items by the study team.				
	Incorporate the suggestion from the experts and result of preliminary field test to determine the final items.		√		√
Reliability and validity examination	A second pilot test is conducted to examine the psychometric characteristics of the items including internal consistent reliability, test-retest reliability, inter- and intra-rater reliability, content validity, criterion validity and construct validity.		√	√	√
Field test to assess feasibility	The controlled environment can evaluate key logistical aspects of implementation in the field. It can also analyze how scores differed by item and by raters, and examine different ways of analyzing and presenting scores.	√	√	√	√

*Note*: The developing steps are referred to the most internationally utilized tools.

Among six steps to construct the instrument, all self-constructed tools carried out “establishment of scoring criteria” and “field test to assess feasibility.” In addition to the Athletic Ability Test Scale of Urban Community Children, other tools wholly followed the steps in “original item selection” and “reliability and validity examination.” The CDCC and the Gross Motor Development Assessment Scale showed information on “pretest of initial items” and “determination of final items,” but the Athletic Ability Test Scale of Urban Community Children and the Evaluation System of Sports Ability skipped the two steps.

## Discussion

### Features of prevalent MSAT in Chinese preschoolers

#### International assessment tools

The adoption of the international instrument includes back-translation of the test manual to ensure accurate capture of assessment items, preliminary test to explore the suitability, necessary examination of the psychometric property, item selection, and standardization on a fully representative sample [53, 54]. These steps make it possible to make full of the international tools and obtain the objective test results.

Any assessment tool would be useless if it were not valid and reliable [55]. Even if all the assessments are carried out in China, environmental and participant characteristics can affect the reliability and validity of the same tool. However, the composite reliability, convergent validity, and discriminant validity of the most widely used tool, TGMD-2, was verified just in one city [39]. The other studies using this tool in different regions did not examine reliability and validity before the formal trial. Besides, none of the included studies conducting item selection reported the reliability and validity data since the psychometric characteristics of the trimmed tool may change. Lacking information on psychometric properties may lead to the quality and applicability of this research assessment tool.

The item selection lacks a more scientific process and reason [56]. Among three studies using partial items in the TGMD-2 [40–42], two of them interpreted that the specific purposes for item selection were to estimate coordination skills of joints and the effect of ball games on object control skills, respectively [40, 41]. They offered information about the aims of selection and which items were determined. However, these studies did not explain why these items were selected and whether the selection was validated. The arbitrary selection of items is problematic as it will threaten the comparability of test results across studies and make it difficult to replicate findings [57].

Moreover, the sequence of the preliminary investigation and item selection is missing. Only Dai followed the usual procedure to conduct the pilot test, then to determine the final items by considering the test results and experts’ suggestions together [40]. The other studies identified the test items without a pilot test. Besides, no study considered the sport habits of children while selecting test items, which may affect the test results. For example, when the TGMD-3 was introduced to Germany, two-hand striking was removed because children did not know how to do it [58].

When the international MAST was used in China, the researchers paid less attention to the translation. Specifically, only one study in this review mentioned the translation process, and its translation was administered by an independent translator [35]. Potentially biased translation occurs when the instrument is translated and administered by only one translator [59]. The shortage of rigorous translation processes and professional translators leads to misunderstanding of the instrument.

Furthermore, the testers with appropriate backgrounds play an essential role during the assessment. In this review, five studies using international tools described the source of testers, but most of them had no information about how testers were trained and whether the practice was carried out before formal tests. The testers’ lack of training and practice may lead to implementation problems during the assessment process and affect the data reliability. Besides, only two studies reported inter-rater reliability [35, 45], whereas inter-rater reliability can validate evaluation results.

In this review, the TGMD-2 appears in a large proportion of studies because of its good applicability. It is the most widely used tool globally and has good reliability and validity under different cultural backgrounds [59–63]. Besides, the TGMD-2 has been translated into Chinese already [59], making it the first choice of MSAT in China. Other tools, such as the BOT-2, the Movement Assessment Battery for Children (MABC), and the Motor-Proficiency-Test for children between four and six years of age (MOT 4–6), are not used regularly or never used, which might result from their late investigation, including the lack of Chinese version. For example, compared with other countries that assessed the MABC in 1996, China used it until 2001 [64, 65].

Moreover, the MABC-2 is often used to identify children with motor impairment, such as developmental coordination disorder (DCD) [14]. Severe neurological and sensory injuries such as cerebral palsy occur in 10% to 15% of preterm/low birth weight infants, but almost 50% of preterm infants develop motor impairment such as DCD [66]. Therefore, several studies in China have used the MBC-2 to investigate the effects of preterm birth on motor performance in pre-school-age children [67, 68], and few studies used the MBC-2 to observe motor performance in normal preschoolers.

In this study, we found that a few international tools were used. The investigation of these tools was incomplete in terms of translation, verification of reliability and validity, item selection, and administration.

#### Self-constructed tools

Most self-constructed tools are not widely used in China. Specifically, the majority are applied in one city or district, and the number of subjects was less than 1,000 except for the CDCC [46]. Furthermore, many self-constructed tools are developed by individual or research teams with little supports from the government or institutions, leading to limited generalizability and application in one district. Therefore, the sample size is too small and regional to guarantee the representativeness of subjects in terms of China’s geographic diversity. Combined with the application of the international tools described before, simply using the various international tools with good reliability and validity is easier and more convenient for researchers instead of committing substantial resources to develop a national tool. However, there is a strong need to construct a Chinese localized MSAT because of the cultural differences in motor development between Chinese and Western populations [69].

The test content of Chinese self-constructed tools is unbalanced since it focuses on assessing gross motor skills. Although the CDCC has one item, and the Athletic Ability Test Scale of Urban Community has two items to assess fine motor skills, the number of gross motor skills items is more than fine motor skills items. The Evaluation System of Sports Ability and the Gross Motor Development Assessment Scale does not measure fine motor skills. While several fine motor skill tools are referenced in the international context, cultural differences exist in the development progress. Unlike the gross motor skills assessment’s concern about muscles and joints, fine motor skills assessment involves other factors such as visual memory, making it difficult to consider the unidimensionality and equal interval of scoring [69].

The examination of reliability and validity is not comprehensive in self-constructed tools. Generally, the assessment evaluation includes inter-rater reliability, test-retest reliability, and construct validity [25]. However, the Athletic Ability Test Scale of Urban Community Children, the CDCC, and the Evaluation System of Sports Ability did not test the inter-rater reliability. In addition, the Athletic Ability Test Scale of Urban Community Children and the Evaluation System of Sports Ability did not examine the construct validity. The inter-rater reliability reflects the extent to which the data collected are correct representations of the variables measured. Constructing validity represents the extent to which the measurement tool matches what we want to measure [70, 71].

In some included studies, the presentation of instruction was missing. For example, the Athletic Ability Test Scale of Urban Community Children and the Evaluation System of Sports Ability did not provide instructions for readers, which may confuse future researchers who intend to use the same tool.

In this review, we found that the usage range of self-constructed tools is limited in China. In addition, the development process is generally complete but lacks detailed information such as instruction.

### Evaluation on developing steps of the self-constructed tools

The development of assessment tools includes generating original items, setting scoring criteria, testing statistical properties, determining final items, examining assessment principles (validity and reliability), and field tests (see Table 4). Compared with the international tools, the development of the CDCC and the Gross Motor Development Assessment Scale is relatively complete. However, the development of other self-constructed tools is still in the initial stage because it lacks the necessary content and procedures during the development process.

The process of item determination is incomplete in self-constructed tools. Although most of the self-constructed tools followed the procedure in item generation, half of the tools just determined the final items through consulting experts. They missed the steps to test the difficulty and suitability and add or delete unfit items, which violated the principles of comprehensiveness, objectivity, continuity, and comparability of the item selection [50, 51]. In terms of tools that carried out the “pretest of initial items” and “determination of final items,” the videotaping and writing importance response scale were missed, which are important steps to ensure the content construct validity of the instrument.

Inter-rater reliability, test-retest reliability, construct validity, and content validity are examined to test its applicability when all items are settled. However, the Athletic Ability Test Scale of Urban Community Children did not verify its reliability and validity. The Evaluation System of Sports Ability only verified the scale reliability and skipped the pilot test procedure to change items that affect the psychometric property. Moreover, criterion-related validity also appears to explore how well the new tool agrees with other tools for assessing the same behavior and predicts the outcome [72]. But only the CDCC verified the criterion-related validity with the Standdord-Bibent test of intelligence subscale. The absence of criterion validity in self-constructed tools may be due to the general lack of gold standards [73].

The self-constructed tools adopted the assessment contents of the most used international tools in the establishment of its subscales and test items. For example, the Gross Motor Development Assessment Scale contains object control and locomotor subscales, similar to the TGMD-2. Likewise, balance and manual dexterity in the MABC-2 can be found in the CDCC and the Athletic Ability Test Scale of Urban Community Children. On the other hand, the self-constructed instrument adopted some items and subscales but did not wholly copy the international tools. For instance, the subtest of the Gross Motor Development Assessment Scale refers to the subtest in TGMD-3. Still, the Gross Motor Development Assessment Scale adds posture control to the subtest because the high relation between posture control and self-perception, anxiety, and depression can reflect the correlation between motor and psychological development in children [49, 74].

### Recommendations

#### The application of international assessment tools

Even though some international tools have been examined to be reliable and valid for Chinese preschoolers, cross-cultural differences appeared on some items as the development of motor skills is affected by environmental background and societal attitudes. Therefore, unless the tool has been vigorously evaluated on a fully representative sample of a national subject, it is crucial to examine the reliability and validity when the tool is used in different areas.

The cross-cultural translation process is necessary since it clarifies scientific test instruction to the source instrument [75]. Therefore, if the instrument has no Chinese version yet, the translation procedure should be carried out and presented in the article. The double-back reverse independent translation is always adopted, which involves four bilingual professional translators to complete the independent translations. The translators have no access to the original version of the assessment. After the translation process, all translators will compare the translated version with the original version and revise it [59]. No matter which method is adopted, it is necessary to guarantee the original version’s linguistic, conceptual, operational, and metric equivalence through professional translators and proof panels [76, 77].

Generally, international tools such as adding and deleting items are revised after the preliminary investigation and examination of the psychometric property. Future research should show which items are tested and interpret why these items are determined and the scientific support behind the reason, including the preliminary test results, the advice from experts, and the psychometric quality of the tool. For example, the reliability and validity of items “walking heels raised” and “drawing trail” of the MABC-2 were lower than other items, which suggested that the two items need refinement when the MABC-2 is used on Chinese children [35]. In addition, the scoring criteria of items need to make relative adjustments according to the sport habits in children.

The standardization establishes consistent procedures, including observation, administration, equipment, and scoring rules. However, future research should show more details about the equipment and observation of assessment as they are not found in the included studies. Besides, the recruited testers should be able to master the basic knowledge of the theory and principles of assessments, professional training, and related experience of children [25].

Last but not least, future studies should expand the sample size into developing standardized versions and establishing national norms after the preliminary investigation, which will help in interpreting and comparing the test results.

#### The development of Chinese self-constructed tools

In the stage for the development of the assessment tool, an initial examination of reliability and validity must be integrated into the construction plan. Firstly, the development details, including why the tool is developed, how items are determined, and how to rank a child’s performance, should be described clearly to strengthen its reliability and validity. Apart from the primary examination, including inter-rater reliability, test-retest reliability, construct validity, and content validity, other tests such as concurrent validity should be used to verify the quantitative and qualitative aspects of motor skills. This step is necessary for constructing the new instrument. Besides, the item selection should be guided by a conceptual and strong framework and examined by construct validity [78].

When a new tool is constructed, the item selection process should be clear and organized. Based on the test purpose, the review of related assessment should be conducted to find the eligible and similar items. For example, many existing tools regard items in the National Standard of Physical Fitness as reference. Then questionnaires should be used to seek expert advice for initial items. After determining original items, the pilot test is necessary to test the difficulty, discrimination, and correlation coefficient between the item and its dimension. During the pilot test, videotapes are found effective in clarifying procedural uncertainties and resolve problems of interpretation in training. The pilot test results will be combined with expert judgment to delete or add items to the tool. The test items can combine assessment with the game to enhance enjoyment and avoid the abnormal behaviors of children in front of strangers and unfamiliar test equipment [50].

When the final draft of the instrument is settled, it can be carried out in the field. Therefore, it is necessary to commit immense resources to collect data on a fully representative sample of a national subject and put the test to a thorough analysis, helping us establish national norms and promote generalization. Noticeably, the subjects from different areas exercise and play differently, have different school experiences, and have quite different gender stereotypes. The published norms and even the nature of the test items require radical reconsideration. Experts should independently score all the items and suggest a specific change of item. Then the modified instrument will be field-tested with a preliminary sample. The adaptation suggested by the experts and field tests should be incorporated into a comprehensive tool.

Given that current self-constructed tools mainly measure gross motor skills, a comprehensive MSAT aiming to assess fine and gross motor skills is needed in China. Fine motor skills are the set of capacities that form the motor skills system with gross motor skills to improve the physical activity level [79]. The pre-school years are when fine motor skills, including bimanual skills, manual dexterity, object manipulation, and eye-hand coordination, develop rapidly [80, 81]. Therefore, the assessment of fine motor skills can complete the understanding of children’s current motor development level and promote children’s later cognitive development, language, and writing skills [82–84].

## Conclusion

The present study has reviewed prevalent MSAT used in Chinese 3–6 years old children, including international and self-constructed assessment tools. Results indicated that international tools used in China were incomplete in terms of translation, verification of reliability and validity, item selection, and implementation. Furthermore, the test content, validity and reliability, implementation instruction, and generalizability are still lacking in the self-constructed tools. After comparing the current self-constructed tools and the most utilized international tools, recommendations were suggested for the MSAT to modify their application in Chinese pre-school children. Finally, cultural differences in motor development between Chinese and Western populations should be considered when constructing a Chinese localized MSAT.

## Data Availability

The datasets used and/or analysed during the current study are available from the corresponding author on reasonable request.

## Abbreviations

AST-1: Athletic Skills Track
BOTMP: Bruininks–Oseretsky Test of Motor Proficiency
BOT-2: Bruininks-Oseretsky Test of Motor Proficiency, second edition
CAT: Communication Assessment Tool
CDCC: Children’s Developmental Centre of China scale
CMSP: CHAMPS Motor Skills Protocol
DCD: Developmental coordination disorder
KTK: Körperkoordination-Test für Kinder
PA: Physical activities
MABC: Movement Assessment Battery for Children
MABC-2: Movement Assessment Battery for Children-2nd edition
MOT 4-6: Motor-Proficiency-Test for children between 4 and 6 years of age
MSAT: Motor skills assessment tools
MSC: Motor Skill Checklist
PCDI: Preschool Child Development Inventory
PDMS-2: Peabody Developmental Motor Scales, second edition
TGMD-3: Test of Gross Motor Development, third edition
TGMD-2: Test of Gross Motor Development 2nd edition
ZNA-2: Zurich Neuromotor Assessment second edition
